# Maternal nano-titanium dioxide inhalation alters fetoplacental outcomes in a sexually dimorphic manner

**DOI:** 10.3389/ftox.2023.1096173

**Published:** 2023-03-06

**Authors:** Julie A. Griffith, Allison Dunn, Evan DeVallance, Kallie J. Schafner, Kevin J. Engles, Thomas P. Batchelor, William T. Goldsmith, Kimberley Wix, Salik Hussain, Elizabeth C. Bowdridge, Timothy R. Nurkiewicz

**Affiliations:** ^1^ Department of Physiology, Pharmacology, and Toxicology, West Virginia University School of Medicine, Morgantown, WV, United States; ^2^ Center for Inhalation Toxicology, West Virginia University School of Medicine, Morgantown, WV, United States

**Keywords:** titanium dioxide, thromboxane (TXA_2_), prostacyclin, sexual dimoprhism, placental flow

## Abstract

The placenta plays a critical role in nutrient-waste exchange between the maternal and fetal circulations, thus functioning as an interface that profoundly impacts fetal growth and development. The placenta has long been considered an asexual organ, but, due to its embryonic origin it shares the same sex as the fetus. Exposures to toxicant such as diesel exhaust, have been shown to result in sexually dimorphic outcomes like decreased placental mass in exposed females. Therefore, we hypothesize that maternal nano-TiO_2_ inhalation exposure during gestation alters placental hemodynamics in a sexually dimorphic manner. Pregnant Sprague-Dawley rats were exposed from gestational day 10–19 to nano-TiO_2_ aerosols (12.17 ± 1.69 mg/m^3^) or filtered air (sham-control). Dams were euthanized on GD20, and fetal tissue was collected based on fetal sex: whole placentas, placental junctional zone (JZ), and placental labyrinth zone (LZ). Fetal mass, placental mass, and placental zone percent areas were assessed for sex-based differences. Exposed fetal females were significantly smaller compared to their exposed male counterparts (2.65 ± 0.03 g vs 2.78 ± 0.04 g). Nano-TiO_2_ exposed fetal females had a significantly decreased percent junctional zone area compared to the sham-control females (24.37 ± 1.30% vs 30.39 ± 1.54%). The percent labyrinth zone area was significantly increased for nano-TiO_2_ females compared to sham-control females (75.63 ± 1.30% vs 69.61 ± 1.54%). Placental flow and hemodynamics were assessed with a variety of vasoactive substances. It was found that nano-TiO_2_ exposed fetal females only had a significant decrease in outflow pressure in the presence of the thromboxane (TXA_2_) mimetic, U46619, compared to sham-control fetal females (3.97 ± 1.30 mm Hg vs 9.10 ± 1.07 mm Hg) and nano-TiO_2_ fetal males (9.96 ± 0.66 mm Hg). Maternal nano-TiO_2_ inhalation exposure has a greater effect on fetal female mass, placental zone mass and area, and adversely impacts placental vasoreactivity. This may influence the female growth and development later in life, future studies need to further study the impact of maternal nano-TiO_2_ inhalation exposure on zone specific mechanisms.

## 1 Introduction

Adverse intrauterine environments have been shown to influence fetal development in a sex-dependent way, as was classically shown in work examining the Dutch famine at the end of WWII (Gabory et al., 2013). Undernutrition in mid to late gestation caused a sex-based difference, with increased placental thickness in female fetuses compared to males (Roseboom et al., 2011). It was speculated this may be the female placenta’s attempt to compensate for reduced growth by deeper spiral artery invasion (Roseboom et al., 2011). Diseases, such as preeclampsia (PE), can also result in hindered fetal development in a sexually dimorphic manner. It has been suggested that the normal growth of males in PE pregnancies is due to an adaptation in peripheral microvascular tone by the maternal circulation to maintain fetal-placental blood flow, despite disrupted hemodynamics and placental insufficiency (Stark et al., 2006). Female fetuses from a PE pregnancy do not demonstrate altered microvascular function, due to a lack of compensatory peripheral vascular responses, have reduced uteroplacental blood flow, and thusly decreased placental hemodynamics that ultimately decrease fetal female growth and development (Stark et al., 2009). In addition to nutritional or vascular derived disease states affecting fetal growth and development, environmental exposures during gestation may also result in compromised fetal health.

Exposure to certain toxicants can modify placental and fetal growth in a sexually dimorphic manner (Miller et al., 2020). A rat model of inhaled ozone found that fetal females from this study demonstrated adaptive mechanisms to increase nutrient availability to support fetal development, while males did not (Miller et al., 2020). In pregnant mice exposed to diesel exhaust, female offspring in the exposed group had decreased placental mass and crown-to-rump length (Behlen et al., 2021). Exposed fetal females demonstrated increased placental decidua area, lacunae areas, and lipid metabolism signaling (Behlen et al., 2021). While not an inhalation exposure, arsenic exposure *via* drinking water in humans also produces a sexually dimorphic effect, with female placentas having increased levels of the aquaglyceroporin transporter (Winterbottom et al., 2017). This transporter may lead to increased movement of arsenic across the female placenta and elicit the expression of a subset of genes that are female-specific in response to arsenic exposure (Winterbottom et al., 2017). Maternal inhalation of nano-titanium dioxide, a nanomaterial used in building materials and water/air filters (Bowdridge et al., 2019), during gestation has caused decreased female pup mass (Griffith et al., 2022) and decreased male: female sex ratios in early and mid-gestation (Garner et al., 2022b). This exposure paradigm our laboratory has utilized for nano-titanium dioxide (nano-TiO_2_) inhalation exposure is a model for pregnant women working in an occupational setting would experience. This encompasses the time periods when women may not realize they are pregnant through late gestation (Griffith et al., 2022). These studies indicate that maternal environmental exposures affect offspring in a sexually dimorphic manner that appears to be paradigm specific. Adaptations in vascular reactivity, to estrogen stimulatory compounds such as prostacyclin (Sobrino et al., 2010), may be part of the sexual dimorphic responses seen in detrimental *in utero* environments such as improper nutrition, disease, or toxicant exposure.

Prostacyclin (PGI_2_) and thromboxane (TXA_2_), potent vasodilator and vasoconstrictor, respectively, are vital in establishing vascular resistance systemically, and are especially critical to the uterine microcirculation and placental vasculature. In normotensive human pregnancies, umbilical arteries and chorionic plate arteries had decreased PGI_2_ induced-vasodilatory capability (Chaudhuri et al., 1993). A study using human placenta chorionic plate vessels determined the TXA_2_ mimetic, U46619, increased perfusion pressure in normotensive fetal placental circulation *in vitro* (Read et al., 1999). A rat gestational hypoxia model demonstrated that vasoactivity can change in both the uterine circulation and umbilical vein (Aljunaidy et al., 2016). Our lab has demonstrated that maternal nano-titanium dioxide (nano-TiO_2_) inhalation alters the uterine microcirculation and results in increased sensitivity to the vasoconstrictive actions of U46619 (Griffith et al., 2022). Maternal inhalation of nano-TiO_2_ also reduced vasodilation in response to the stable PGI_2_ analog, carbaprostacyclin (Griffith et al., 2022). In conjunction with this, we have also found that maternal nano-TiO_2_ inhalation during gestation results in litter decreased fetal mass (Bowdridge et al., 2019), increased placental mass (Bowdridge et al., 2019), and decreased male: female ratio in early and mid-gestational exposures (Garner et al., 2022b). Further studies expanded on the fetal mass and determined that maternal nano-TiO_2_ inhalation during gestation specifically decreased female pup mass (Griffith et al., 2022). This has led us to hypothesize herein that maternal nano-TiO_2_ inhalation exposure during gestation alters placental hemodynamics and therefore influences fetal health outcomes in a sexually dimorphic manner.

## 2 Materials and methods

### 2.1 Animal model

Timed pregnant Sprague-Dawley (SD; delivered on GD 5–10) rats were purchased from Hilltop Laboratories (Scottdale, PA) and single-housed in an American Association for Accreditation of Laboratory Animal Care (AAALAC) approved facility at West Virginia University (WVU) Health Sciences Center. Rats were housed in a maintained environment: temperature (20–26°C), relative humidity (30–70%), and light-dark cycle (12:12 h). Rats were acclimated for 48–72 h, then randomly assigned to either sham-control (N = 13) or nano-TiO_2_ (N = 14) exposure groups. Rat cages were lined with standard bedding (0.25-inch corncob) and had *ad libitum* access to standard chow (2918X; Envigo, Indianapolis, IN) and water throughout the acclimation and exposure periods.

On GD 20, rats were weighed and then anesthetized with isoflurane gas (5% induction, 2–3.5% maintenance), placed on a warm heating pad, and maintained at a rectal temperature of 37°C. Rats were euthanized *via* thoracotomy and heart removal and then distribution of fetuses within the uterine horns and fetal sex was recorded. Fetal tissue was weighed and grouped according to fetal sex: whole placentas, placental junctional zone, and placental labyrinth zone. All procedures were approved by the WVU Institutional Animal Care and Use Committee.

### 2.2 Nanomaterial

Nano-TiO_2_ powder was obtained from Evonik (P25 Aeroxide TiO_2_, Parsippany, NJ) and is composed of a mixture of anatase (80%) and rutile (20%) TiO_2_. Particle characteristics have previously been determined, including primary particle size (21 nm), specific surface area (48.08 m^2^/g), and Zeta potential (-56.6 mV) (Yi et al., 2013; Stapleton et al., 2018).

### 2.3 Inhalation exposure and aerosol characterization

A high-pressure acoustical generator (HPAG, IEStechno, Morgantown, WV) created nano-TiO_2_ aerosols. Output from the generator was fed into a Venturi pump (JS-60M, Vaccon, Medway, MA) to further de-agglomerate particles. The nano-TiO_2_ aerosol mix enters a whole-body exposure chamber and a personal DataRAM (pDR-1500; Thermo Environmental Instruments Inc., Franklin, MA) samples the air to determine aerosol mass concentration in real-time. Software feedback loops automatically adjust the acoustic energy needed to maintain a stable mass aerosol concentration throughout the exposure. Gravimetric aerosol sampling measurements were conducted with Teflon filters concurrently with the DataRAM measurements to obtain calibration factors. Gravimetric measurements were taken during each exposure to calculate the mass concentration measurement. Real-time aerosol size distributions were measured in the exposure chamber at a target mass concentration of 12 mg/m^3^ *via*: 1) a high-resolution electrical low-pressure impactor (ELPI+; Dekati, Tampere, Finland); 2) a scanning particle mobility sizer (SMPS 3938; TSI Inc., St. Paul, MN); 3) an aerodynamic particle sizer (APS 3321; TSI Inc., St. Paul, MN); and 4) a micro-orifice uniform deposit impactor (MOUDI 115R, MSP Corp, Shoreview, MN). Bedding material was soaked to maintain proper humidity (20–70%). Similar temperature and humidity conditions were maintained in exposure chambers utilized only for sham-control animals, which were exposed to HEPA-filtered air only.

Inhalation exposures were performed for 6 non-consecutive days from GD 10–19 to prevent pregnancy loss. A target concentration of 12 mg/m^3^ was used for late gestation inhalation exposure (Stapleton et al., 2013; Stapleton et al., 2018). For estimation of lung deposition (dose) with nano-TiO_2_ aerosols, equation D = F•V•C•T was used where F is the deposition fraction (10%), V is the minute ventilation (208.3 cc), C is mass concentration (mg/m^3^), and T equals the exposure duration (minutes) (Nurkiewicz et al., 2008; Stapleton et al., 2013). The exposure paradigm (12 mg/m^3^, 6 h/exposure, 6 days) produced a calculated cumulative lung deposition of 525 ± 16 µg (Bowdridge et al., 2022; Griffith et al., 2022) with the last exposure occurring on GD19 24-h prior tissue collection. The calculations represent total lung deposition and do not account for lung clearance (MPPD Software v 2.11, Arlington, VA).

### 2.4 Pressure myography with isolated placentas

Once dams were euthanized and pups per horn count was recorded, the uterus was surgically excised and placed into a dissection dish containing physiological salt solution (PSS, in mmol/L: 129.8 NaCl, 5.4 KCl, 0.5 NaH_2_PO_4_, 0.83 MgSO_4_, 19.0 NaHCO_3_, 1.8 CaCl_2_, 5.5 glucose). The uterus was incised longitudinally, and amnionic sacs were opened to allow for quick identification of fetal sex. Fetal sex and position within the horn was recorded, then the first male and female nearest the cervix were removed with the placenta still attached. The placenta/pup units were placed into dissection dishes with PSS maintained at 4°C and were utilized for placental hemodynamic assessment (Garner et al., 2022a).

The umbilical artery and vein were separated from the umbilical cord. Once the amnionic sac and vitelline vessels were removed, the umbilical vessels were cut as close to the pup as possible (Garner et al., 2022a). The placenta was then closed at the site of implantation with 6–0 silk sutures (AD Surgical, Sunnyvale, CA) and placentas were transferred to an isolated vessel chamber (Living Systems Instrumentation, Burlington, VT) containing 10 ml of oxygenated (21% O_2_/5% CO_2_) 37°C PSS. The umbilical artery was attached to the inflow glass pipette tip and the umbilical vein was attached to the outflow pipette tip (Garner et al., 2022a) using 6–0 silk sutures. Placentas were then pressurized from 0 to 20 mm Hg in 5 mm Hg increments.

Outflow pressure and flow rate were assessed following addition of vasoactive drugs to assess vascular hemodynamics. Endothelium-dependent responses were assessed by acetylcholine (ACh, 1 × 10^−4^ M), application of s-nitroso-N-acetyl-dl-penicillamine (SNAP, 1 × 10^−4^ M) assessed endothelium-independent responses, addition of phenylephrine (PE, 1 × 10^−4^ M) assessed α_1_-adrenergic vasoconstriction, addition of carbaprostacyclin (1 × 10^−10^ M) assessed cyclooxygenase metabolite vasodilation, and application of U46619 (1 × 10^−4^ M) assessed cyclooxygenase metabolite vasoconstriction. Drugs were added to the bath individually to assess outflow pressure and flow rate response. Washes were done between each drug to ensure clearance. Once all drug response were assessed PSS was removed and replaced with Ca^2+^-free PSS to assess passive maximum outflow pressure and flow rate.

### 2.5 Placental histology

Male and female placentas were collected from sham-control (N = 5 per sex) and nano-TiO_2_ (N = 5 per sex) exposed dams. Placentas were perfused with 4% paraformaldehyde and fixed *ex situ* with 4% paraformaldehyde at 4°C overnight. Placental tissue was then cleared with phosphate buffer solution (PBS) and transferred PBS overnight. Tissue was then rapidly frozen *via* isopentane and liquid nitrogen and stored at -80°C. Using a cryostat at -20°C, placentas were sectioned at 10 µm thickness. Sections from the center of the placenta, which provided the largest cross-sectional area, were placed on subbed slides, and stained with hematoxylin and eosin (H&E) following provided protocol instructions (Vector Laboratories, Burlingame, CA). Tissue sections were incubated with hematoxylin for 1 min and eosin for 5 min. Slides were imaged at 10x and analyzed using ImageJ (Xu et al., 2020). Total placental area and percent total area of the junctional and labyrinth zones were determined using an average of measures from three sections per pup.

### 2.6 Immunohistochemical staining of placentas

Male and female placentas from sham-control (N = 5 per sex) and nano-TiO_2_ (N = 5 per sex) dams were collected on GD20. Placentas were perfused and then placed into a 4% paraformaldehyde fixative overnight at 4°C and then transferred to PBS for the following night (Bowdridge et al., 2019). Tissue was then flash frozen and stored at -80°C until sectioned (Bowdridge et al., 2019). Placentas were sectioned at 10 µm through the center of the placenta. Three sections per pup were analyzed. Sections were washed 4 × 5 min with 0.1 M PBS to remove cryoprotectant. Sections were then incubated for 10 min with 1% H_2_O_2_ and washed 4 × 5 min with 0.1 M PBS. Sections were incubated for 1 h at room temperature with 0.1 M PBS, 0.4% Triton-X100 (Sigma-Aldrich, St. Louis, MO, United States of America) and 20% normal goat serum (NGS; Jackson ImmunoResearch Laboratories, Inc., West Grove, PA, United States of America). Sections were then incubated with mouse monoclonal anti-Pan cytokeratin antibody (1:250, F3418; Sigma-Aldrich) (Nteeba et al., 2020) for 24 h at 4°C. Slides were washed 5 × 5 min, and then incubated in mouse monoclonal anti-rat CD163 (1:500, MCA342R; Bio-Rad Laboratories, Hercules, CA, United States of America) (Rosario et al., 2009) for 24 h at four°C. The final day, slides were washed with 0.1 M PBS 3 × 5 min. Then incubated with Alexa555 goat anti-mouse IgG1 (1:200; A21127; Thermo Fisher Scientific Inc., Waltham, MA, United States of America) for 1 h. Slides were washed 4 × 5 min and covered with a coverslip using ProLong Diamond Antifade Mountant with DAPI (Thermo Fisher). Slides were stored in the dark at 4°C until analysis.

### 2.7 statistics

Dam characteristics, such as age, mass, and litter size, were assessed by unpaired *t*-test with Welch’s correction. The remainder of the dam characteristics were assessed by two-way analysis of variance (ANOVA). Fetal mass characteristics and total area of placental zones were analyzed *via* a two-way mixed-effects ANOVA. A two-way mixed effects model was used for assessing point-to-point differences in dose response curves to vascular agonists and increased pressure curves. If statistical significance occurred, then a Tukey *post hoc* test was used for all ANOVA analysis. All data are reported as mean ± SEM, unless otherwise stated. Significance was set at *p* ≤ 0.05.

## 3 Results

### 3.1 Nanoparticle aerosol characteristics

**FIGURE 1:**
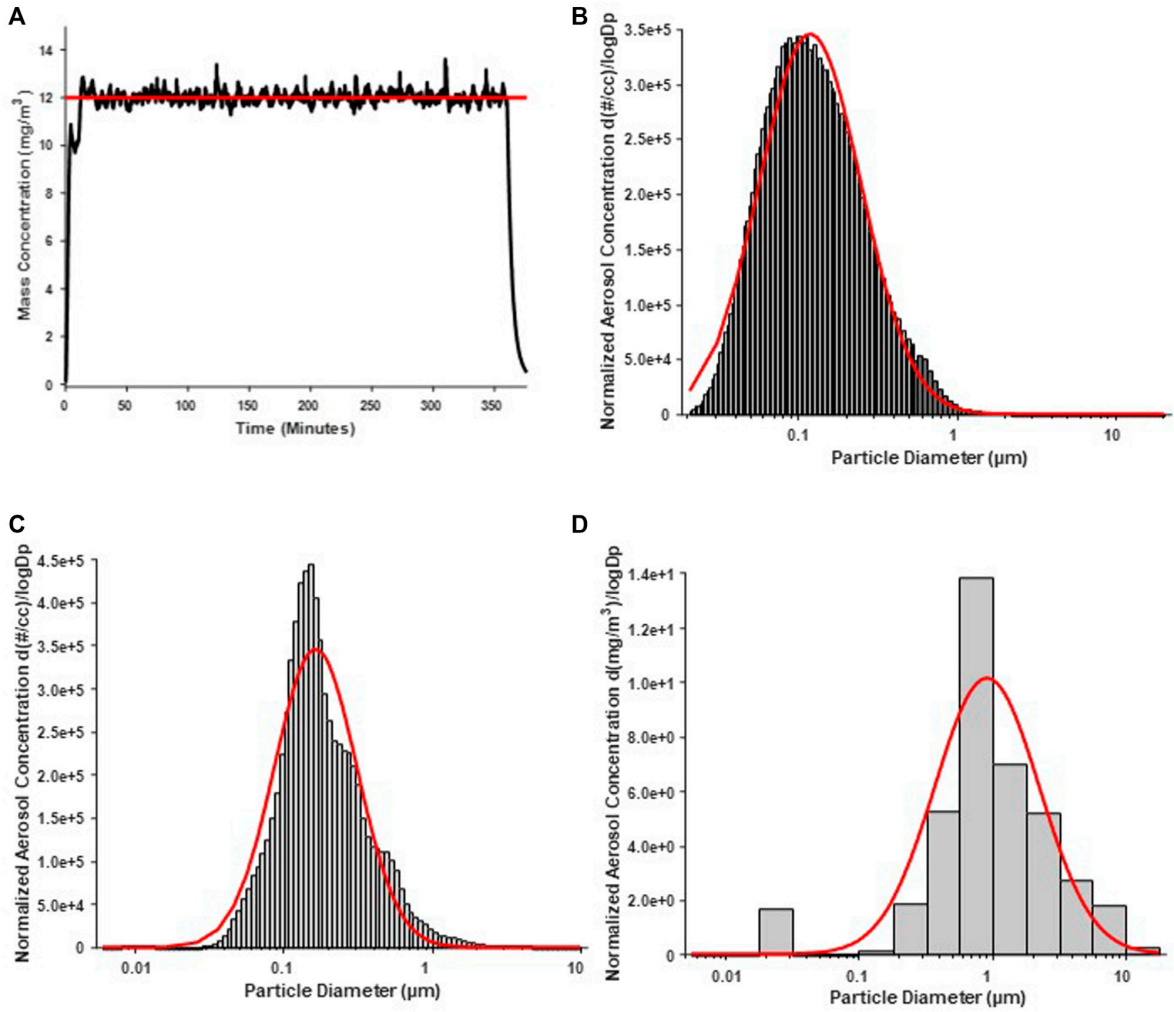
Nano-TiO_2_ aerosol real-time characterizations. Aerosol characterization was monitored and verified during the exposure periods. Red lines indicate size distribution curves for a log normal fit of the size data. **(A)** Program software-controlled aerosol mass concentration over the 6-h exposure paradigm. Real-time nano-TiO_2_ aerosol mass concentration (black line) was maintained near the desired 12 mg/m^3^, the target average concentration (red line). **(B)** Aerosol aerodynamic diameter was assessed by scanning mobility particle sizer (SMPS; light gray) and an aerodynamic particle sizer (APS; dark gray). The particle diameter, determined by SMPS and APS, had a CMD of 118 nm and a geometric standard deviation of 2.10. **(C)** Aerosol aerodynamic diameter was also assessed by a high-resolution electrical low-pressure impactor (ELPI) which had a CMD of 164 nm and geometric standard deviation of 1.89. **(D)** A nano micro-orifice uniform deposit impactor (MOUDI) was used to evaluate aerosol mass size distribution and indicated a mass median aerodynamic diameter (MMAD) of 0.918 µm and a geometric standard deviation of 2.47.

Average real-time aerosol mass concentration over the course of exposures was 12.17 mg/m^3^ with a standard deviation of 1.69 (Figure 1A). SMPS and APS measured the aerosol mobility diameter, which had a count median diameter (CMD) of 118 nm and a geometric standard deviation (GSD) of 2.10 (Figure 1B). ELPI assessment of the aerosol aerodynamic diameter showed, a mass median aerodynamic diameter (MMAD) of 164 nm with a geometric standard deviation of 1.89 (Figure 1C). A Nano Micro-Orifice Uniform Deposit Impactor (MOUDI 115R, MSP Corp, Shoreview, MN) was utilized to measure mass size distribution, which had a mass median aerodynamic diameter (MMAD) of 0.92 µm and a GSD of 2.47 (Figure 1D). The morphology of the nano-TiO_2_ agglomerates has been previously characterized extensively with electron microscopy (Abukabda et al., 2019; Bowdridge et al., 2019).

### 3.2 Pregnant rat and litter characteristics

Dams displayed no significant differences in age, litter size, or fetal sex on GD20 between groups (Table 1). There was a significant decrease in dam mass on GD20 in nano-TiO_2_ exposed dams (N = 14) compared to sham-control (N = 13; Table 1).

**TABLE 1:** Dam and litter characteristics include dam age (days), mass (grams), litter size, pup distribution across horns, fetal sex distribution, and resorptions distributions across horns. N is the number of dams. Data are mean ± SEM. *, *p* ≤ 0.05 vs sham-control.

Exposure	N	Dam age (d)	Dam mass (g)	Litter size (pup number)	Fetal horn distribution (pup number)		Sex distribution (pup number)		Resorptions distribution (number of sites)	
					Left	Right	Male	Female	Left	Right
Sham-control	13	70.2 ± 2.2	358.4 ± 9.3	13.2 ± 0.6	6.7 ± 0.4	6.5 ± 0.6	6.5 ± 0.3	6.2 ± 0.5	0.9 ± 0.09	0.55 ± 0.21
Nano-TiO_2_	14	71.9 ± 1.6	**330.8 ± 4.7** *	12.6 ± 0.6	7.0 ± 0.5	5.6 ± 0.6	6.0 ± 0.4	5.4 ± 0.2	0.08 ± 0.08	0.23 ± 0.17

**FIGURE 2:**
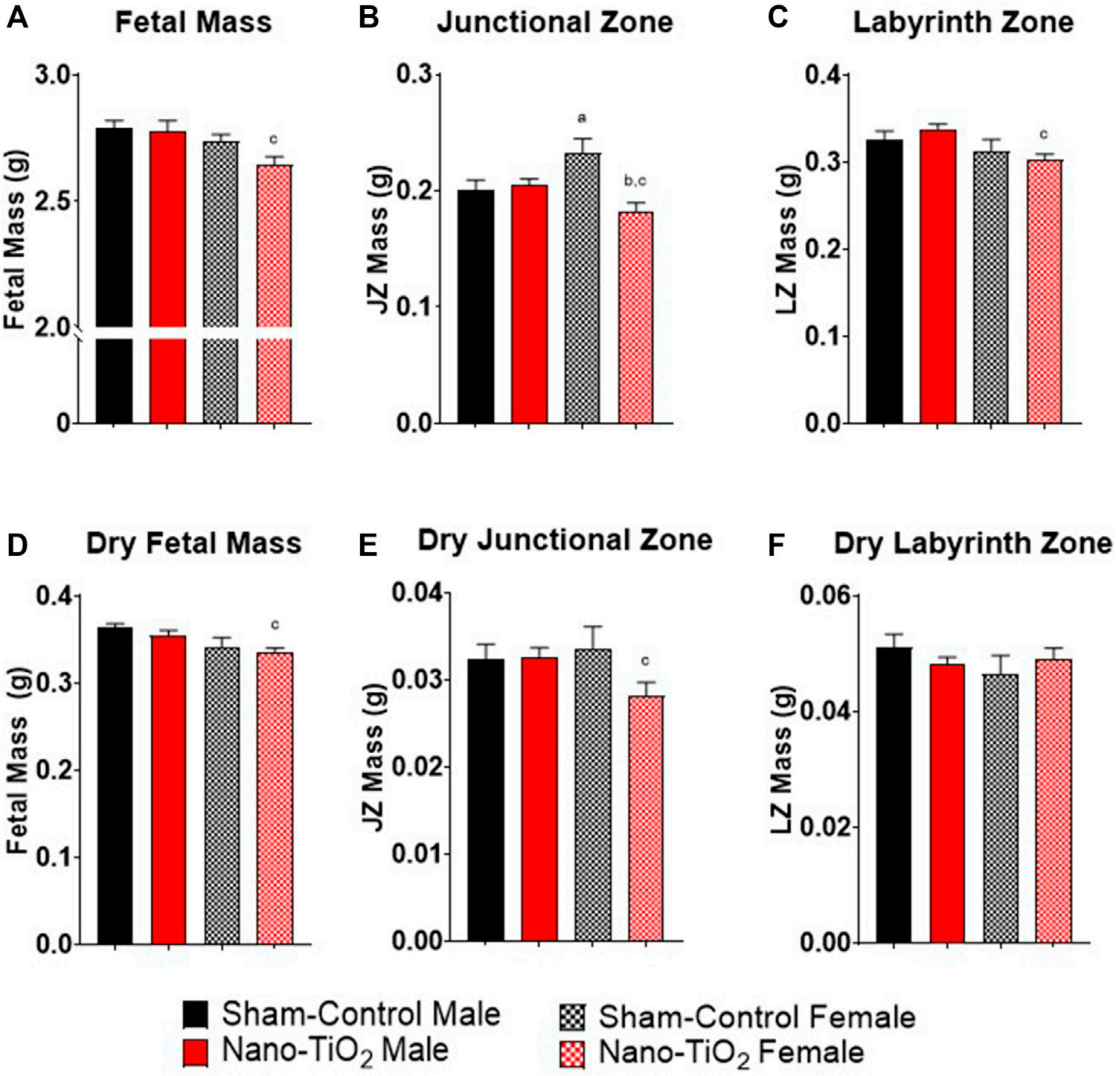
Fetal Pup and Placental Zone Mass. Fetal and placental mass were recorded based on fetal sex on GD 20 (N = 13, sham-control fetal male and female, and N = 14, nano-TiO_2_ fetal male and female). **(A)** Wet fetal mass. **(B)** Wet junctional zone mass. **(C)** Wet labyrinth zone mass. **(D)** Dry fetal mass. **(E)** Dry junctional zone mass. **(F)** Dry labyrinth zone mass. a, *p* ≤ 0.05 vs sham-control fetal male. b, *p* ≤ 0.05 vs sham-control fetal female. c, *p* ≤ 0.05 vs nano-TiO_2_ fetal male.

Fetal pup and placental zone mass after nano-TiO_2_ inhalation exposure during gestation were assessed according to fetal sex to determine any sexually dimorphic outcomes (Figure 2). Maternal nano-TiO_2_ inhalation exposure significantly decreased fetal female wet mass (2.65 ± 0.03 g) compared to the nano-TiO_2_ fetal males (2.78 ± 0.04 g; Figure 2A). The nano-TiO_2_ exposed fetal female mass decreased compared to the sham-control fetal female mass (2.74 ± 0.03 g). The placenta is composed of two core regions, the junctional zone (JZ) and the labyrinth zone (LZ). The JZ (Figure 2B) presented sex-based differences in mass and an effect of exposure seen only in the females. Sham-control fetal females (0.23 ± 0.01 g) had significantly larger JZ compared to sham-control fetal males (0.20 ± 0.01 g). Nano-TiO_2_ exposed fetal female JZ wet mass (0.18 ± 0.01 g) was significantly decreased compared to sham-control fetal females (0.23 ± 0.01 g) and nano-TiO_2_ exposed fetal males (0.20 ± 0.04 g). The wet LZ mass (Figure 2C) of nano-TiO_2_ fetal females (0.30 ± 0.01 g) was significantly smaller compared to nano-TiO_2_ fetal males (0.34 ± 0.01 g). This indicates that maternal nano-TiO_2_ inhalation exposure during gestation has a greater effect on fetal female pup and placenta mass.

Dry mass was also measured to discern if wet mass differences were driven by water content. Dry fetal mass (Figure 2D) was significantly decreased in nano-TiO_2_ fetal females (0.33 ± 0.01 g) compared to the nano-TiO_2_ male counterparts (0.35 ± 0.01 g). The dry sham-control fetal males (0.36 ± 0.01 g) tended to be larger than the dry fetal sham-control females (0.34 ± 0.01 g). Dry JZ mass (Figure 2E) was also significantly decreased in nano-TiO_2_ fetal females (0.028 ± 0.001 g) compared to nano-TiO_2_ fetal males (0.033 ± 0.001 g). There were no significant differences in dry LZ mass across treatment groups (Figure 2F). This provides evidence that nano-TiO_2_ exposure during gestation causes structural mass changes, not based on water content.

### 3.3 Sexually dimorphic placental hemodynamics

**FIGURE 3:**
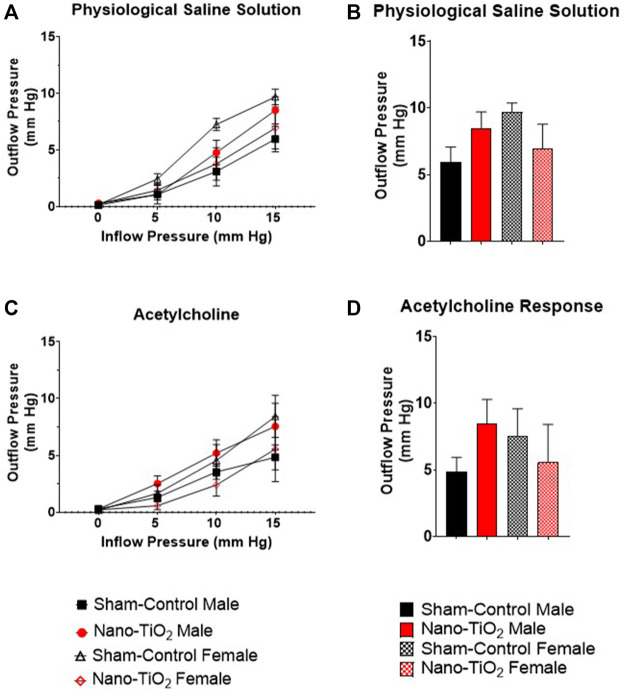
Placental Outflow Hemodynamics. Outflow pressure readings were recorded in conjunction with increased inflow input. **(A)** Outflow pressure in the presence of physiological saline solution (PSS). **(B)** Maximum outflow response in the presence of PSS. **(C)** Outflow pressure in the presence of the endothelium-dependent vasodilator, ACh. **(D)** Maximum outflow response in the presence of ACh. Sham-control fetal male, n = 5-6, nano-TiO_2_ fetal male, n = 5-7, sham-control fetal female, n = 4-5, and nano-TiO_2_ fetal female, n = 5-6.

**FIGURE 4:**
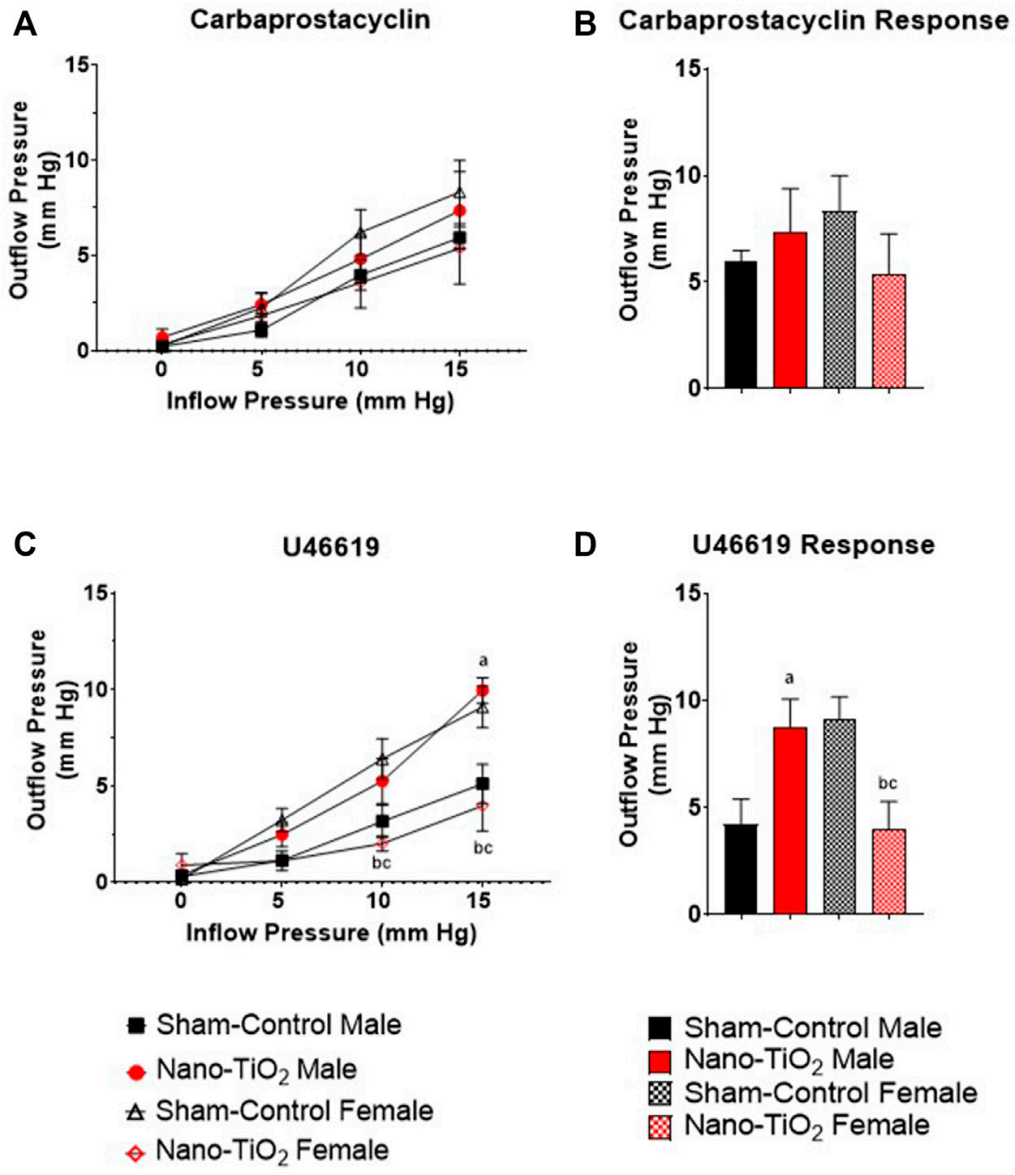
Placental Cyclooxygenase Metabolites Outflow Hemodynamics. Outflow pressure readings were recorded with increased inflow input. **(A)** Placental outflow pressure in the presence of carbaprostacyclin, a stable PGI_2_ agonist. **(B)** Maximum outflow response to carbaprostacyclin. **(C)** Outflow pressure with U46619, the TXA_2_ mimetic. **(D)** Maximum outflow pressure response in the presence of U46619. Sham-control fetal male, n = 5-6, nano-TiO_2_ fetal male, n = 5-7, sham-control fetal female, n = 4-5, and nano-TiO_2_ fetal female, n = 5–6. a, *p* ≤ 0.05 vs sham-control fetal male. b, *p* ≤ 0.05 vs sham-control fetal female. c, *p* ≤ 0.05 vs nano-TiO_2_ fetal male.

Placental outflow pressure for sham-control fetal males (n = 5–6), sham-control fetal females (n = 4–6), nano-TiO_2_ fetal males (n = 5–7), and nano-TiO_2_ fetal females (n = 5–7) was measured to assess vascular resistance within the perfused tissue. Placentas were incubated in the presence of PSS, ACh, carbaprostacyclin, and thromboxane and outflow pressure was assessed (Figures 3, 4). Responses were also determined following exposure to SNAP, PE, and Ca^2+^-free PSS (Supplementary Figure S1).

Placental responses to physiological saline solution (PSS) were assessed to test baseline outflow pressure (Figures 3A, B), which was not significantly different amongst treatments. Placental outflow pressure response to the endothelium-dependent vasodilator, ACh (Figures 3C, D) were not significantly different amongst treatments. Responses to carbaprostacyclin, a cyclooxygenase vasodilator (Figures 4A, B), were also not significantly different between groups. The thromboxane mimetic, U46619 (Figures 4C, D), resulted in sham-control fetal females to have increased outflow compared to sham-control fetal males at 15 mm Hg inflow pressure (9.10 ± 1.07 mm Hg vs 5.11 ± 1.02 mm Hg). Placentas of nano-TiO_2_ exposed fetal females had significantly decreased outflow pressure (3.97 ± 1.30 mm Hg) compared to sham-control fetal females (9.10 ± 1.07 mm Hg) and nano-TiO_2_ exposed fetal male placentas (9.96 ± 0.66 mm Hg). Nano-TiO_2_ exposed fetal male placentas also had significantly increased outflow compared to sham-control fetal males (9.96 ± 0.66 vs 5.11 ± 1.02 mm Hg, respectively). There were no significant differences between groups for outflow pressures when incubated with PE, SNAP, or Ca^2+^-free PSS (Supplementary Figure S1A–C). This indicates that maternal nano-TiO_2_ inhalation exposure during gestation results in modified placental hemodynamics that are specific to thromboxane.

**FIGURE 5:**
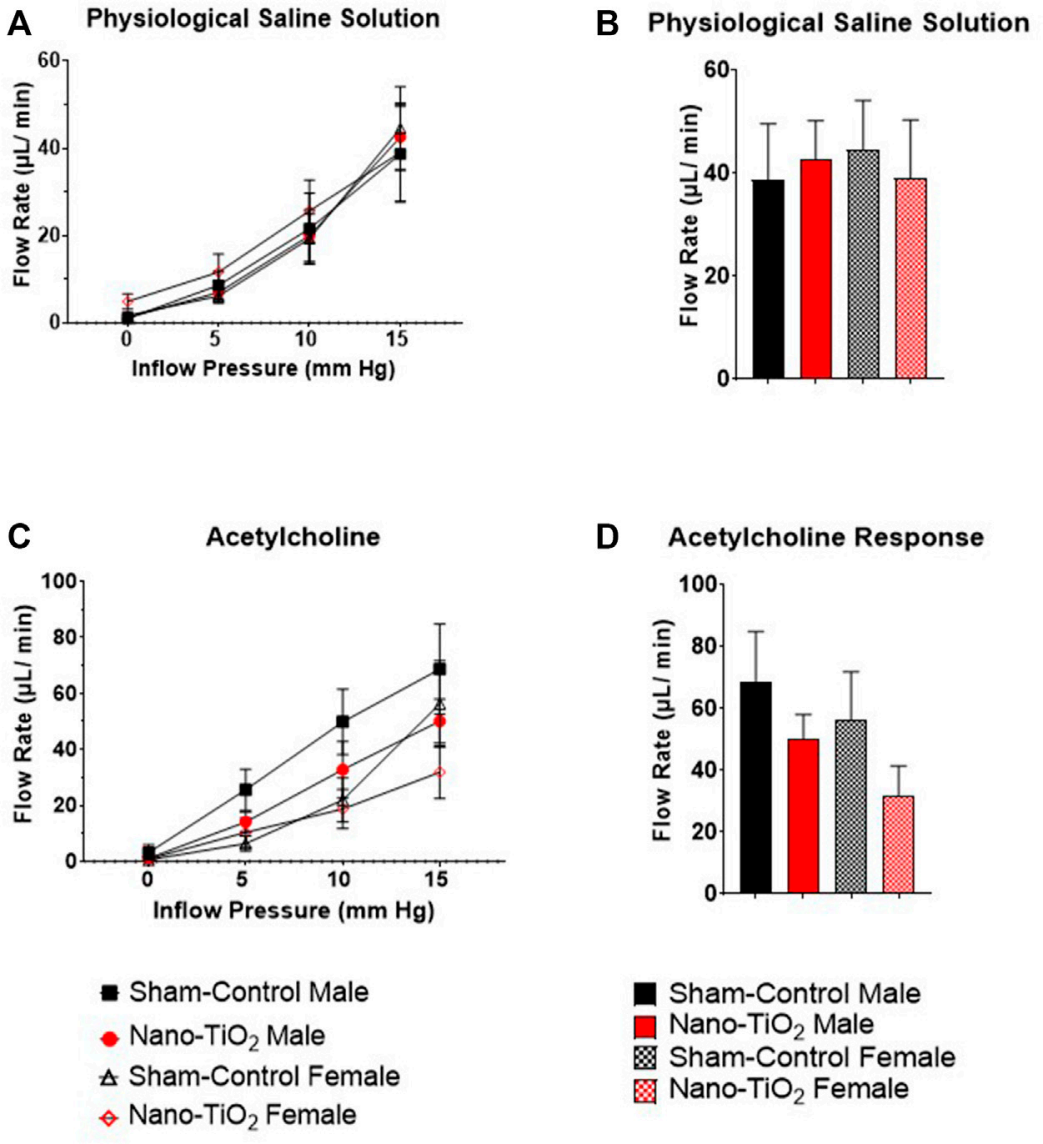
Placental flow rate hemodynamics. Flow rate was calculated and recorded throughout experiment as inflow pressure was increased stepwise manner. **(A)** Flow rate in PSS bathe. **(B)** Maximum flow rate response in PSS. **(C)** Flow rate of placentas in the presence of the ACh, an endothelium-dependent vasodilator. **(D)** Maximum flow rate response across increased pressure. Sham-control fetal male, n = 5-6, nano-TiO_2_ fetal male, n = 5-7, sham-control fetal female, n = 4-5, and nano-TiO_2_ fetal female, n = 5-6.

**FIGURE 6:**
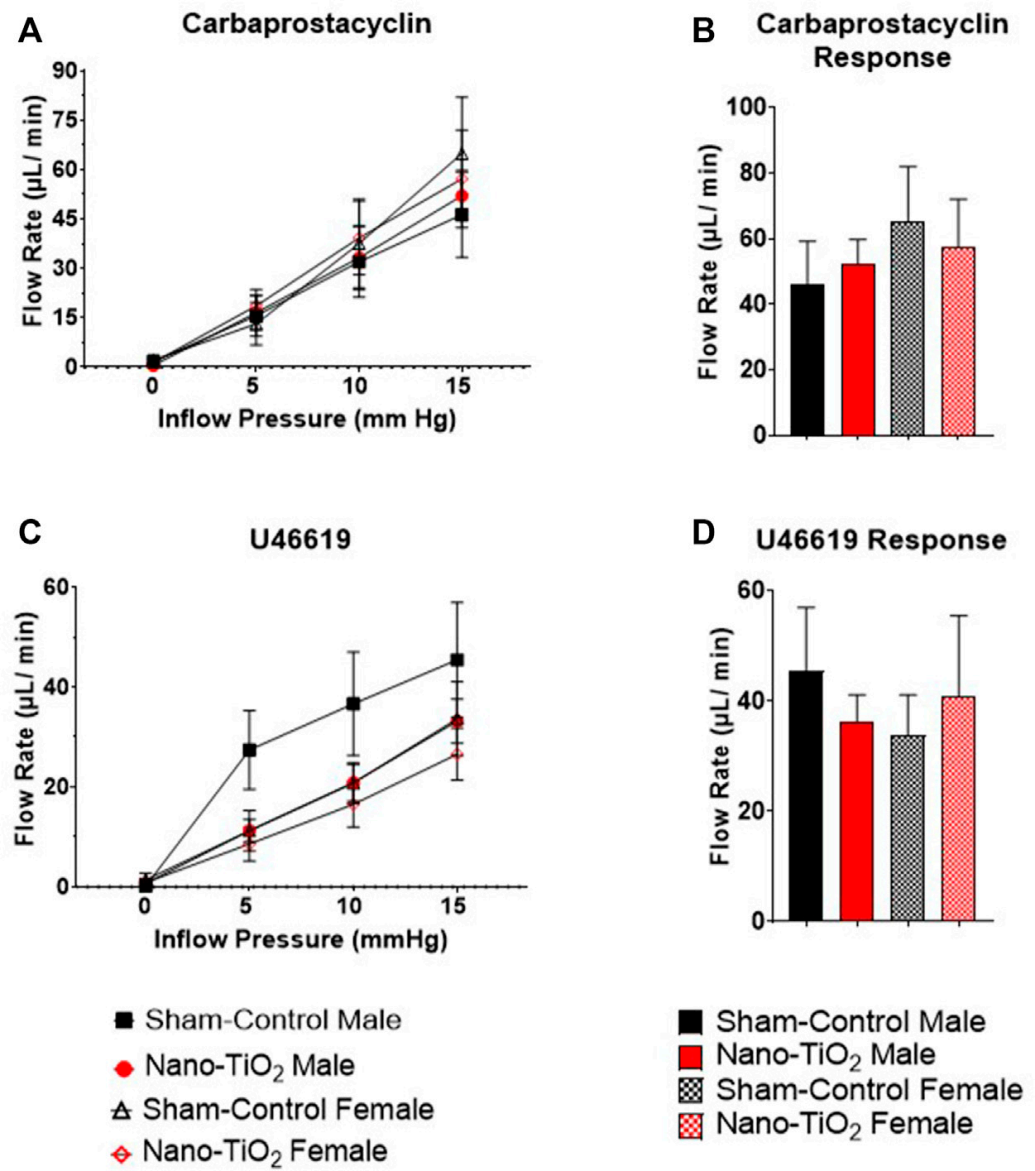
Placental Cyclooxygenase Metabolites flow rate hemodynamics. Flow rate was calculated and recorded during inflow pressure increases in a stepwise manner. **(A)** Placenta flow rate in the presence of carbaprostacyclin, a stable PGI_2_ agonist, across increased inflow pressure. **(B)** Maximum flow rate in response to carbaprostacyclin. **(C)** Placenta flow rate with U46619, a TXA_2_ mimetic, added to the bathe. **(D)** Maximum flow rate response to U46619. Sham-control fetal male, n = 5-6, nano-TiO_2_ fetal male, n = 5-7, sham-control fetal female, n = 4-5, and nano-TiO_2_ fetal female, n = 5-6.

Placental flow rates were also assessed for each group to determine hemodynamic responses to inflow pressure and vasoactive drugs. There were no significant differences for placenta flow rates across groups when incubated with PSS, ACh, PGI_2_ analog, carbaprostacyclin, and the TXA_2_ mimetic, U46619 (Figures 5A–D and Figures 6A–D). Placenta flow rates did not demonstrate significant differences between groups when incubated with PE, SNAP, or Ca^2+^-free PSS (Supplementary Figure S2A–C).

### 3.4 Placental histology & immunohistochemistry

**FIGURE 7:**
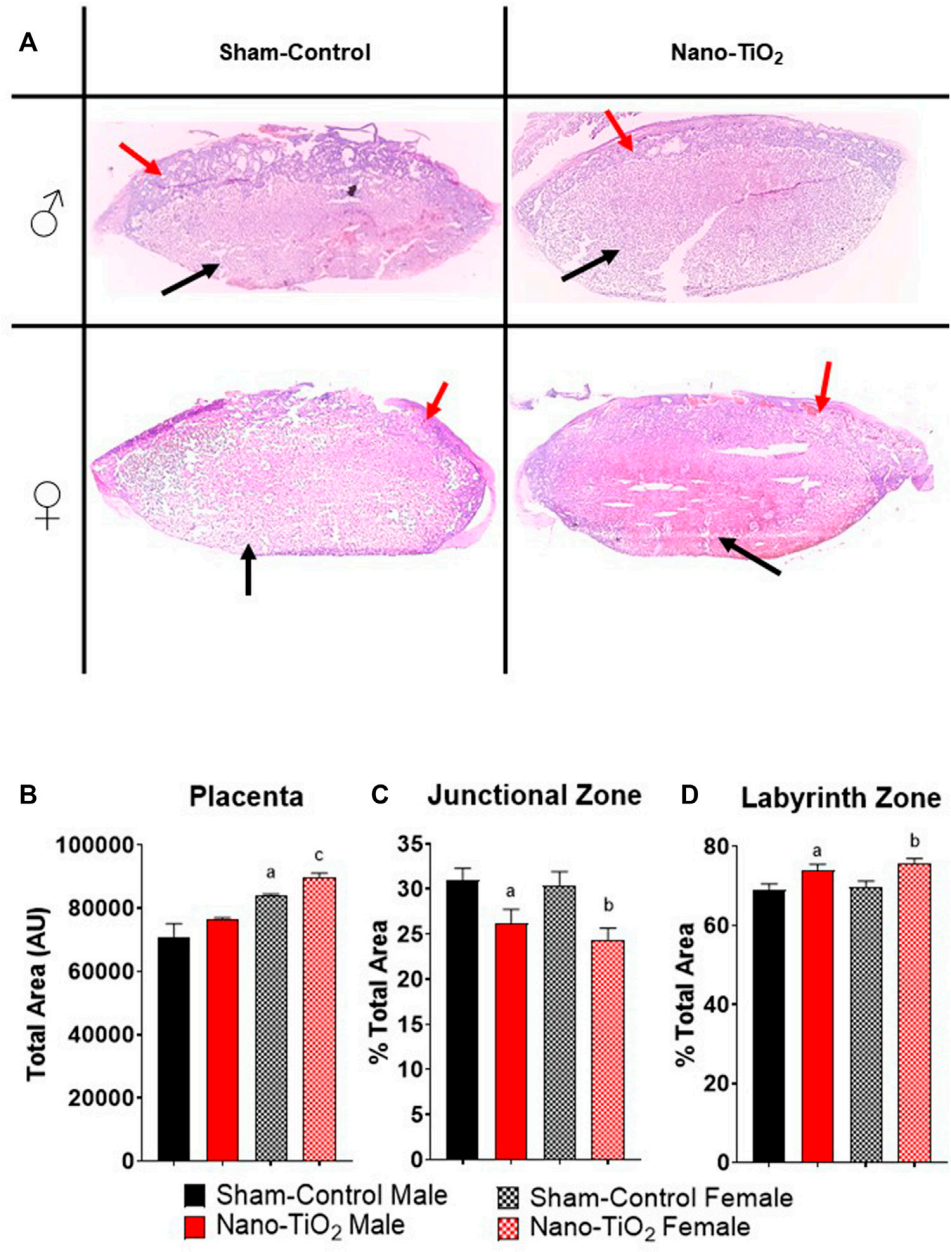
Placental Histology. Total placental zone areas were assessed after H&E staining to determine anatomical and structural differences after maternal nano-TiO_2_ inhalation exposure. **(A)** Representative images of placenta histology are depicted for each group. **(B)** Total area of the placenta. **(C)** Percent total area of the junctional zone (JZ). **(D)** Percent total area of the labyrinth zone (LZ). Sham-control males (n = 5), sham-control females (n = 5), nano-TiO_2_ males (n = 5), and nano-TiO_2_ females (n = 5). a, *p* ≤ 0.05 vs sham-control fetal male. b, *p* ≤ 0.05 vs sham-control fetal female. c, *p* ≤ 0.05 vs nano-TiO_2_ fetal male.

Placentas were collected for each group (sham-control males and females (n = 5/sex); nano-TiO_2_ males and females (n = 5/sex)) to assess differences in placental JZ and LZ area and anatomy between fetal sex and exposure paradigm. A representative image of the placenta histology is shown in Figure 7A. Total placenta area was assessed, in which there was a significant increase in area size for sham-control female compared to sham-control male (84,130 ± 3834 AU vs 69,956 ± 3660 AU; Figure 7B). Nano-TiO_2_ female area was also significantly increased compared to nano-TiO_2_ male total placenta area (89,697 ± 6141 AU vs 76,558 ± 4272 AU; Figure 7B). The percent JZ area was significantly decreased for nano-TiO_2_ males (26.15 ± 1.59%) compared to sham-control males (30.93 ± 1.37%; Figure 7C). Nano-TiO_2_ females also had a significantly decreased JZ area compared to sham-control females (24.37 ± 1.30% vs 30.39 ± 1.54%; Figure 7C). There was no significant difference between fetal sex within their exposure group (Figure 7C). Total LZ area is highlighted in Figure 7D. Nano-TiO_2_ males had a significant increase in LZ area compared to sham-control males (73.85 ± 1.59% vs 69.07 ± 1.37%). Nano-TiO_2_ females (75.63 ± 1.30%) had a significant increase of LZ area compared to sham-control females (69.61 ± 1.54%; Figure 7D). There was not a significant difference for fetal sex within their exposures. This indicates that not only does maternal nano-TiO_2_ inhalation exposure during gestation change placental mass, but results in placental area changes as well.

**FIGURE 8:**
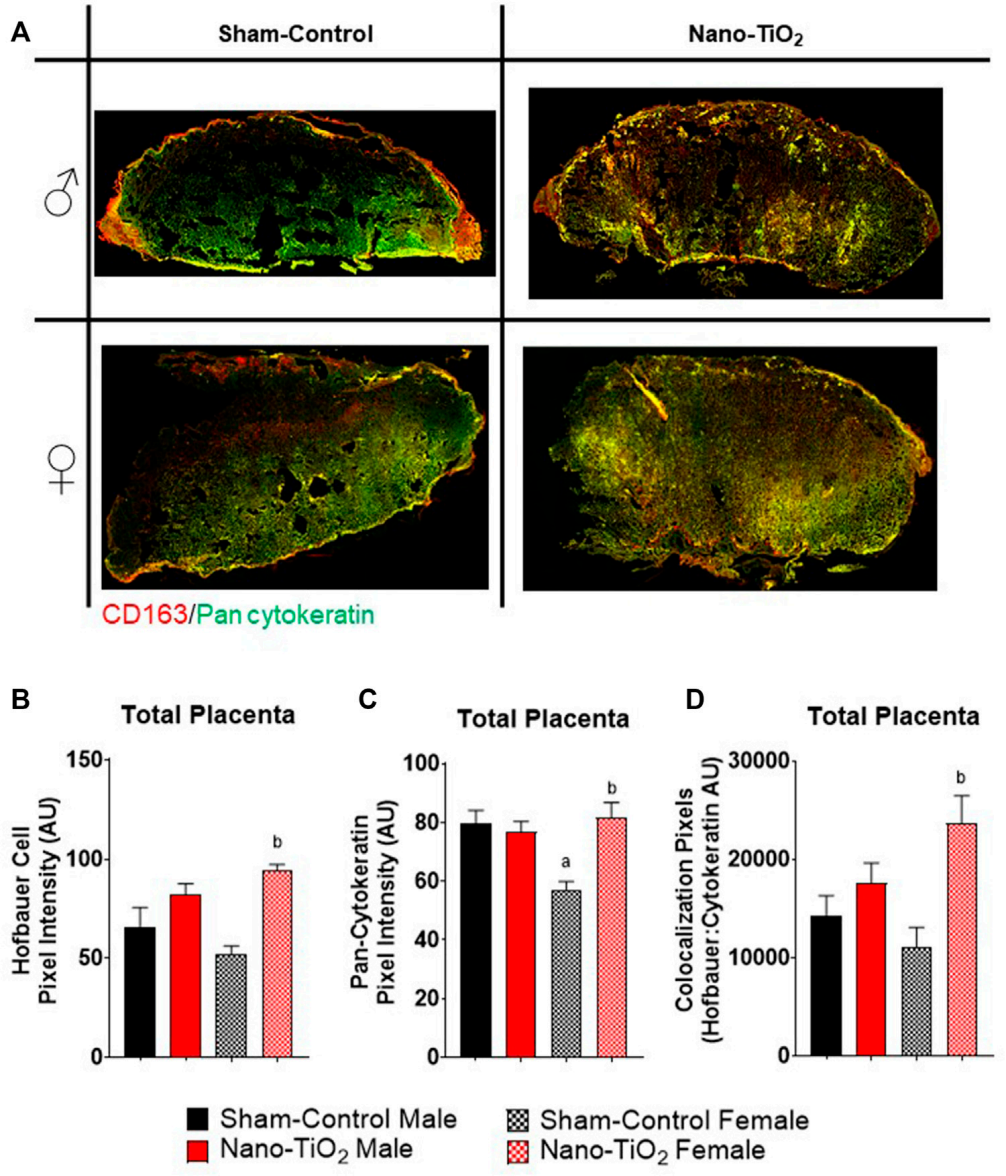
Whole Placental Immunohistochemistry. Total placental fluorescence intensity and colocalization of anti-CD163 and anti-pan cytokeratin was assessed after maternal nano-TiO_2_ inhalation exposure. **(A)** Representative placental images for each group. **(B)** Total placental Hofbauer cell pixel intensity. **(C)** Total placental pan-cytokeratin pixel intensity. **(D)** Total placental colocalization of Hofbauer: Pan-cytokeratin. Sham-control males (n = 5), sham-control females (n = 5), nano-TiO_2_ males (n = 5), and nano-TiO_2_ females (n = 5). a, *p* ≤ 0.05 vs sham-control fetal male. b, *p* ≤ 0.05 vs sham-control fetal female.

Additionally, a subset of placentas was used to quantify Hofbauer cell (CD163; macrophages specific to gestation) and trophoblast cell (anti-Pan cytokeration; placental lineage cells), along with their co-localization. A representative image is provided in Figure 8A. Hofbauer cell pixel intensity for the total placenta is depicted in Figure 8B. There was a significant increase in fluorescent intensity for nano-TiO_2_ females compared to sham-control females (93.41 ± 3.05 AU vs 52.80 ± 6.67 AU; Figure 8B). Trophoblast cell fluorescent intensity was significantly decreased in sham-control female (57.12 ± 3.84 AU) compared to sham-control males (80.17 ± 7.90 AU) and compared to nano-TiO_2_ females (81.18 ± 6.53 AU; Figure 8C) in the total placenta. Colocalization of Hofbauer and trophoblast cells is depicted in Figure 8D. Co-localization was significantly increased in nano-TiO_2_ females compared to sham-control females (23,387 ± 3,172 AU vs 11,293 ± 1,896 AU). Maternal nano-TiO_2_ inhalation exposure during gestation results in modified cellular composition of the placentas.

**FIGURE 9:**
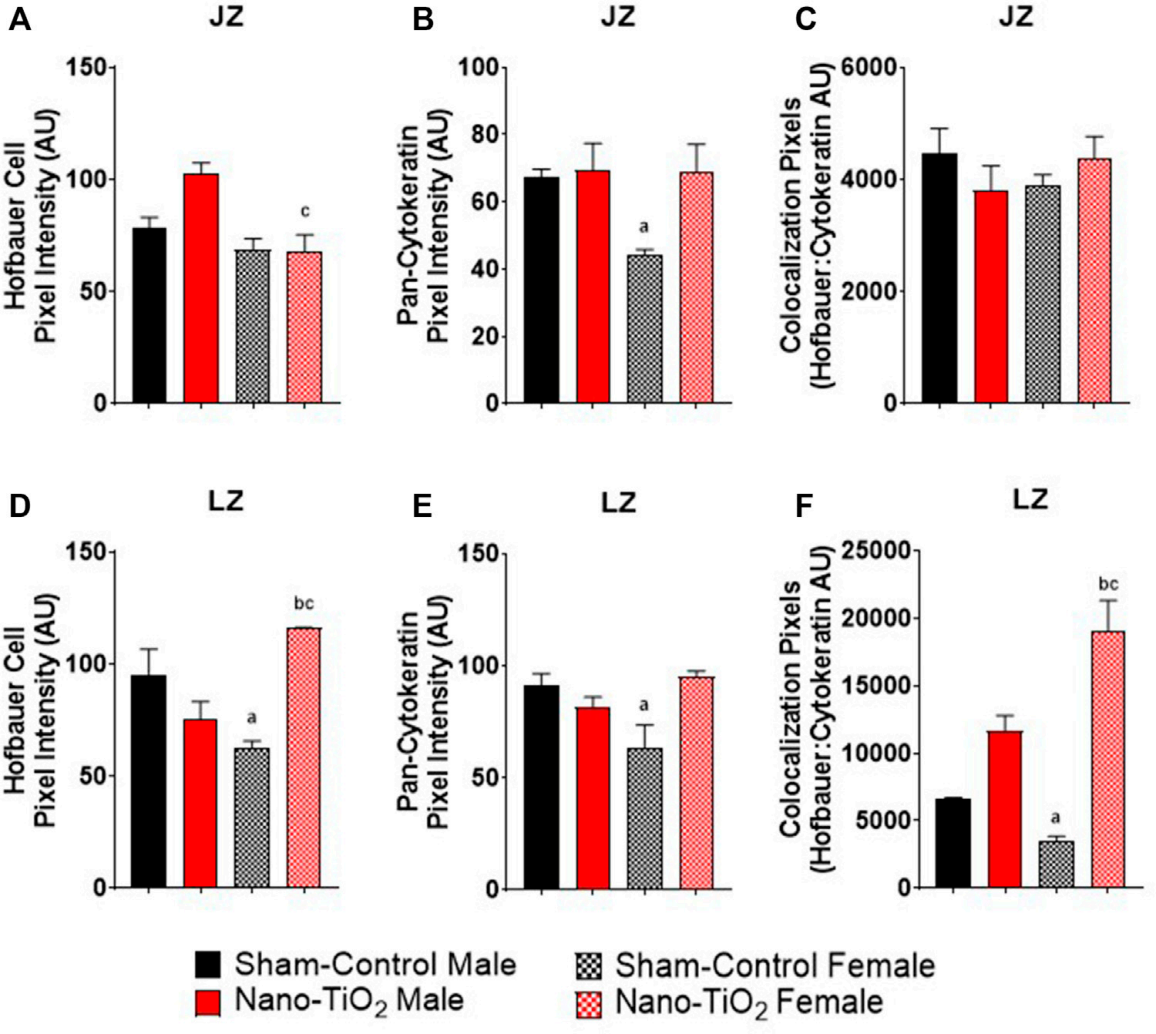
Placental Zone Immunohistochemistry. Placental fluorescence intensity and colocalization of anti-CD163 and anti-pan cytokeratin in each placenta zone. **(A)** Junctional zone (JZ) pixel intensity of anti-CD163 (Hofbauer cell). **(B)** JZ pixel intensity for pan-cytokeratin. **(C)** JZ colocalization for CD163: pan-cytokeratin. **(D)** Labyrinth zone (LZ) pixel intensity for Hofbauer cells. **(E)** LZ pixel intensity for pan-cytokeratin. **(F)** LZ colocalization for CD163: pan-cytokeratin. Sham-control males (n = 5), sham-control females (n = 5), nano-TiO_2_ males (n = 5), and nano-TiO_2_ females (n = 5). a, *p* ≤ 0.05 vs sham-control fetal male. b, *p* ≤ 0.05 vs sham-control fetal female. c, *p* ≤ 0.05 vs nano-TiO_2_ fetal male.

Immunohistochemistry staining was also evaluated based on JZ and LZ fluorescent intensity and colocalization, which is shown in Figure 9. Hofbauer cells significantly decrease in nano-TiO_2_ females compared to nano-TiO_2_ males (66.47 ± 5.08 AU vs 103.1 ± 5.75 AU). Nano-TiO_2_ males tended to have increased CD163 intensity compared to sham-control males (78.65 ± 5.91 AU; *p* = 0.06; Figure 9A). Pan-cytokeratin intensity for JZ was significantly increased in sham-control males compared to females (67.24 ± 5.21 AU vs 44.43 ± 2.59 AU; Figure 9B) and nano-TiO_2_ female (67.38 ± 9.43) tended to have increased compared to sham-control females (*p* = 0.07). Colocalization of CD163 and pan-cytokeratin was assessed in the JZ, in which there was no significant difference (Figure 9C).

Within the LZ, there was a significant decrease for CD163 intensity of sham-control females compared to males (62.00 ± 12.80 AU vs 94.74 ± 15.00 AU; Figure 9D). Nano-TiO_2_ females (116.1 ± 7.869) had significantly increased CD163 intensity compared to sham-control females (62.00 ± 12.80) and nano-TiO_2_ males (75.61 ± 6.58 AU). LZ pan-cytokeratin fluorescence was significantly decreased for sham-control female compared to males (64.65 ± 7.47 AU vs 91.55 ± 10.23 AU; Figure 9E) and nano-TiO_2_ females (95.19 ± 7.19 AU) tended to have increased staining compared to sham-control females (*p* = 0.08). Within the LZ, there was a significant decrease of colocalization for sham-control female (3,415 ± 819.8 AU) compared to sham-control male (6,513 ± 719.8 AU) and nano-TiO_2_ female (18,706 ± 2,802 AU; Figure 9F). There was also a significant increase for colocalization for nano-TiO_2_ female compared to nano-TiO_2_ male (18,706 ± 2,802 vs 11,631 ± 11,577). The placenta zones of the exposed fetuses are also impacted with changes in their cellular composition, and thus may change their functionality.

## 4 Discussion

The primary aim of this project was to determine if maternal nano-TiO_2_ inhalation exposure during gestation alters placental vascular reactivity and fetal growth in a sexually dimorphic manner. Herein, we demonstrated that maternal nano-TiO_2_ gestational inhalation exposure produces placental dysfunction in a sex-dependent manner. While female fetuses have the greatest impact, with decreased placental size and area, fetal growth, and placental hemodynamic capabilities, these are likely adaptations to preserve fetal life. Our laboratory has also previously demonstrated decreased male to female ratio in early and mid-gestation inhalation exposures (Garner et al., 2022b). Males are more susceptible to fetal loss due to external maternal stress during gestation (Kraemer, 2000). Additionally, maternal disease (like Diabetes Mellitus) may affect male fetal congenital development and perinatal outcomes (Evers et al., 2009; García-Patterson et al., 2011). It appears that females are more adaptable in hostile environments to ensure they survive gestation, but these adaptations may be to their detriment later in life.

*In utero* perturbations can result in fetal intrauterine growth restriction (IUGR), which is a risk factor for many adult diseases such as cardiovascular disease (CVD), diabetes, dyslipidemia, hypertension, metabolic syndrome, or renal diseases later in life (Menendez-Castro et al., 2018). Insults that result in a hostile gestational environments that cause diseases later in life is part of the Barker hypothesis, widely referred to as the developmental origins of health and disease (DOHaD) (Barker, 1990; Barker and Martyn, 1992). In this study, we observed a significant decrease in fetal mass for the nano-TiO_2_ exposed fetal females compared to nano-TiO_2_ males (Figure 2A) which was anticipated as this has been previously shown (Griffith et al., 2022). Modification of blood flow or nutrient exchange to the fetus can have different impacts on the progression of fetal growth between sexes. In a gestational guinea pig model, it was found that early-onset hypoxia caused both male and female mass to decrease, but late-onset hypoxia caused only female mass to decrease compared to sex-matched controls (Thompson et al., 2020). Hypoxic models are important as they indicate modifications in blood flow and vascular resistance changes to increased oxygen delivery to critical organs (Heinonen et al., 2016). Intrauterine growth restriction (IUGR), as seen in our study and guinea pig hypoxia study (Thompson et al., 2020), has a strong association with impaired fetal blood flow (Laurin et al., 1987), thus leaving fetuses to attempt to adapt to this hostile gestational environment. Fetal growth can be impacted by toxicant exposures, in a sexually dimorphic manner and these perturbations can be exasperated by direct toxic effects on the placenta.

Toxicant exposures can affect total placental mass, placenta zone mass and area of placental zones. Herein, placental zone mass (Figures 2B, C) and placenta zone areas (Figures 7C, D) changes occurred after nano-TiO_2_ exposure during gestation. Placental perturbations were most pronounced in the nano-TiO_2_ exposed females, which had decreased JZ and LZ mass, decreased JZ area, and increased LZ area. Decreased JZ mass and area could lead to modifications in hormone production and increased LZ area results in modifications to placental nutrient-waste exchange capabilities in a sex-dependent manner (Gårdebjer et al., 2014). Studies of diet restriction, in mice have demonstrated decreased fetal mass (Belkacemi et al., 2009; Coan et al., 2010; Connor et al., 2020), decreased JZ and LZ mass (Belkacemi et al., 2009), and decreased JZ volume (Coan et al., 2010) or area (Schulz et al., 2012; Connor et al., 2020). Undernutrition in mice has also been reported to increase LZ area or volume (Coan et al., 2010; Schulz et al., 2012) and make up a larger proportion of the placenta. These studies came to similar conclusions, that while the overall placenta mass was decreased, the increased LZ area is an attempt to compensate for nutrient restriction and preserve fetal growth (Coan et al., 2010; Schulz et al., 2012). Changes in the mass and area of the JZ and LZ are important to fetal development, however the cellular composition of these zones is equally as important. These changes could be due to cell proliferation or hypertrophy and can result in functional changes in the fetoplacental unit.

A hostile *in utero* environment during gestation may cause the placenta to go through modifications in zone size, mass, area, and volume, as discussed above, but it may also result in cellular composition changes preserve fetal life. Indeed, we observed changes in the cellular composition of placentas in our exposure model. Further, these changes occur in a sexually dimorphic manner in the whole placenta (Figures 8B, C) and in the placenta zones (Figures 9A–E, H). A reduced uteroplacental perfusion pressure (RUPP) mouse model for PE during pregnancy found that RUPP surgery on E14.5 resulted in altered proliferation and differentiation LZ trophoblast makers (Natale et al., 2018). At E16.5, there was an increase in trophoblast and endothelial proliferation markers within the LZ of RUPP placentas (Natale et al., 2018). The JZ of RUPP placentas had a constant trophoblast giant cell (TGC) population, shrinking spongiotrophoblast relative to placenta size, unlike controls which decreased their TGC population over gestation (Natale et al., 2018). It was proposed in this study that the altered trophoblast proliferation was due to hypoxic conditions, which has been demonstrated to trigger trophoblast proliferation *in vitro* (Caniggia et al., 2000; Natale et al., 2018). This is important because increased trophoblast proliferation, may indicate increased fetal blood space area (Natale et al., 2018), to help increase nutrient-waste exchange. The Hofbauer cell marker, CD163, has been shown to preferentially localize near fetal vessels and trophoblasts and are found within the placenta throughout the majority of gestation (Swieboda et al., 2020). Hofbauer cells are still not fully understood, but their function has been shown to be perturbed in diseases like chronic villitis or villitis of unknown etiology (VUE), in which proliferation of Hofbauer cells is seen (Reyes and Golos, 2018). In these disease states, the Hofbauer cells exhibit more inflammatory phenotypes, which is actually thought to cause more placental damage (Reyes and Golos, 2018). In this model, it is likely the trophoblast cells are increasing within the LZ, much like the Hofbauer cells, and both are functioning to compensate for the nutrient restriction and aid to preserve fetal life and growth. Our lab has previously reported that gestational nano-TiO_2_ inhalation exposure alters maternal uterine radial arteriole vascular reactivity (Bowdridge et al., 2019; Garner et al., 2022b; Griffith et al., 2022). Therefore, it is likely that the changes in fetal female JZ and LZ placenta mass and area are changing in response to the toxicant exposure and the upstream alterations that are occurring on the maternal side of the vasculature to preserve fetal life and growth.

**FIGURE 10:**
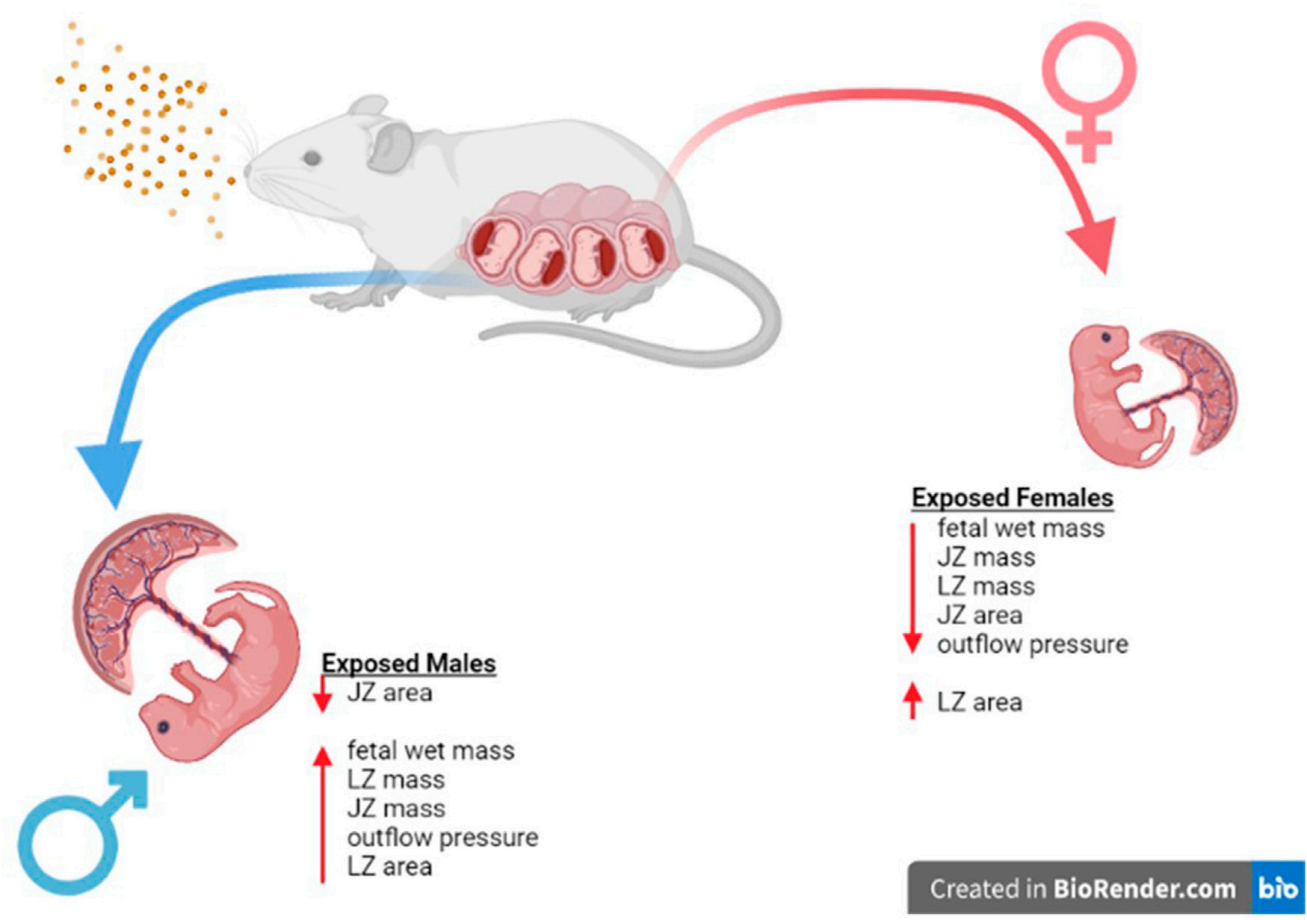
Sex Based Placental Outcomes Summary. Maternal nano-TiO_2_ exposure during gestation results in sexually dimorphic outcomes in the placental and fetal tissue at GD 20. Females undergo more placental changes including decreases in mass (JZ and LZ) and area (JZ) when compared to males. These resulting changes may be responsible for the decreased fetal mass seen in females compared to their male counterparts at GD 20, and these changes have been shown to result in health deficits for the females into adulthood (Bowdridge et al., 2022).

Adaptations within the placental structure and cellular composition are not the only way that fetal life preservation can be achieved in perturbed uterine environments. The maternal side of the vasculature, such as the uterine radial arterioles, have been shown to have reduced vasoreactivity in the presence of vasoactive compounds like prostacyclin or thromboxane (Griffith et al., 2022). Additionally, the placentas from nano-TiO_2_ exposed dams have increased reactive oxygen species production rate (Bowdridge et al., 2022). Increased reactive oxygen species, like H_2_O_2_, has been shown to increase TXB_2_, the stable TXA_2_ metabolite, production in hypertensive rat mesenteric arterioles (Gao and Lee, 2001). This study demonstrates that nano-TiO_2_ exposed placentas also have modified placenta outflow in the presence of thromboxane agonist, U46619 (Figures 4C, D). Exposed fetal female placentas have decreased outflow pressure in the presence of U46619 compared to sham-control females and nano-TiO_2_ fetal males. Understanding the importance of this requires understanding the fetoplacental unit and the flow of nutrient-waste exchange by the umbilical cord (Figure 10). The umbilical vein (outflow from the placenta) carries the nutrient-rich oxygenated blood to the fetus while the umbilical artery (inflow toward the placenta) carries deoxygenated, nutrient-deprived blood to the placenta (Wang and Zhao, 2010). With this in mind, ultrasound and doppler flow measurements visualize and record measurements of umbilical artery, in which greater flow from the fetus to the placenta reflects a healthier fetus, that utilizes more nutrients for increased metabolic processes, growth, and development (Wang and Zhao, 2010). In conjunction with this, reduced umbilical vein blood flow is associated with low fetal birthweight (Wang and Zhao, 2010). In this model, the exposed female placentas demonstrate a decreased outflow pressure in the presence of a highly vasoconstrictive compound, U46619. The decreased outflow pressure reflects elevated placenta resistance in the presence of U46619. It is possible that these placentas have adapted to decrease their responsiveness to TXA_2_ to preserve fetal growth and ultimately prevent fetal death. Females are smaller at GD 20 (Griffith et al., 2022) (Figure 2A) and this same pattern of diminished growth persists up to 8-week (data not published). Additionally, adult females (∼10–11 weeks of age) exposed to nano-TiO_2_ *in utero* will also have smaller pups at GD 20 (Bowdridge et al., 2022) and have decreased plasma estrogen levels, which could be due to the decreased JZ area and mass seen in this study. In studies of mouse maternal nano-TiO_2_ inhalation exposure during gestation we found that fetal hearts had decreased cardiac output and increased LV mass (Kunovac et al., 2019). Young adult mice from this same exposure paradigm demonstrated decreased systolic radial displacement (Hathaway et al., 2017), decreased ejection fraction and fractional shortening (Kunovac et al., 2019). This indicates that maternal exposure to nano-TiO_2_ inhalation during gestation may cause fetal cardiac dysfunction that reaches into adulthood. As such, the fetal females from exposed dams are impacted to a greater extent, as shown by decreased fetal mass, decreased JZ area and mass, increased LZ area, and decreased outflow pressure in the presence of TXA_2_ mimetic. These adaptations may be compensatory mechanisms in impacted females that support gestational survival, growth, and reproduction later in life.

In conclusion, this project sought to determine if maternal nano-TiO_2_ inhalation exposure during gestation alters fetal growth, placental size, trophoblast invasion, and placental vasoactivity in a sexually dimorphic manner. Our exposure paradigm provides evidence that maternal nano-TiO_2_ inhalation exposure had a greater, and lasting, impact on fetal females regarding mass, placental zone mass, zone area, as well as placental vasoactivity. The modifications reported may be physiological adaptations for the fetal females to guarantee survival after maternal nano-TiO_2_ inhalation exposure, but at what cost? Future studies should investigate the impact of maternal nano-TiO_2_ inhalation exposure on zone specific mechanisms: H_2_O_2_ levels, production of TXA_2_ and PGI_2_, and this interaction in a sexually dimorphic manner. This would clarify if H_2_O_2_ and redox metabolites are driving these changes seen based on fetal sex.

## Data Availability

The original contributions presented in the study are included in the article/Supplementary Material, further inquiries can be directed to the corresponding author.
