# Supplementary figures and images for: Maternal nano-titanium dioxide inhalation alters fetoplacental outcomes in a sexually dimorphic manner
Source: Front Toxicol. 2023 Mar 6;5:1096173. doi: 10.3389/ftox.2023.1096173 (PMC10025460; doi:10.3389/ftox.2023.1096173)

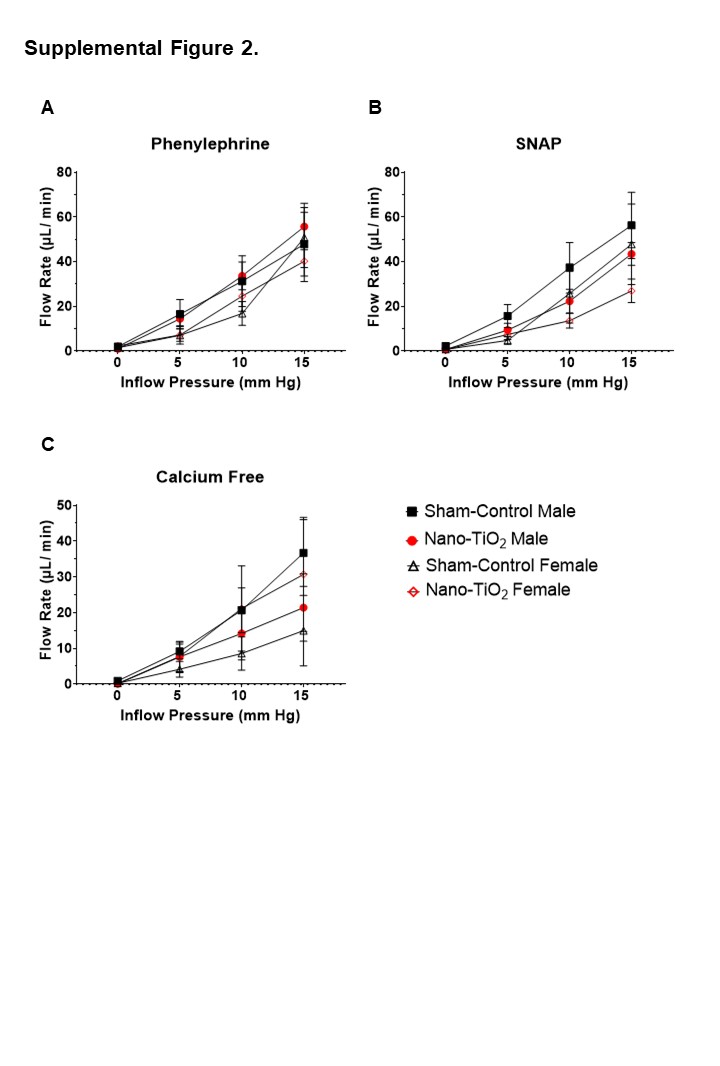

Supplement: Supplementary file 1 [file Image2.jpg]

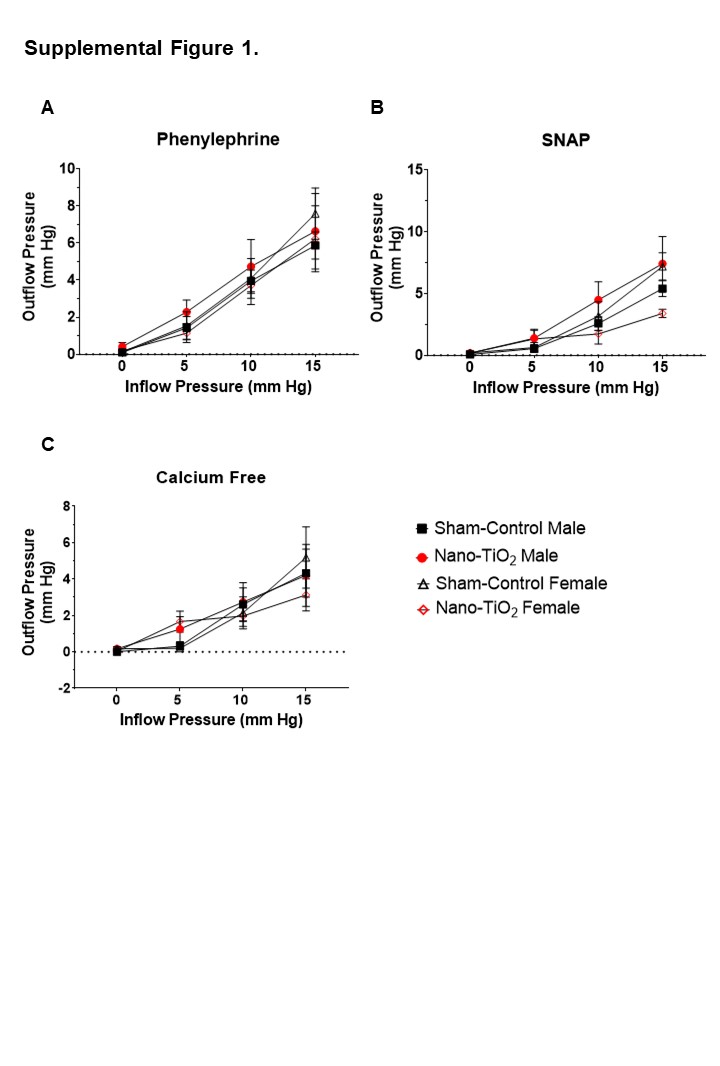

Supplement: Supplementary file 2 [file Image1.JPEG]
